# First report of polymorphisms in *MTRR, GATA4, VEGF, and ISL1* genes in Pakistani children with isolated ventricular septal defects (VSD)

**DOI:** 10.1186/s13052-021-01022-7

**Published:** 2021-03-23

**Authors:** Sumbal Sarwar, Farah Ehsan, Shabana, Amna Tahir, Mahrukh Jamil, Saleem Ullah Shahid, Asim Khan, Shahida Hasnain

**Affiliations:** 1 Institute of Microbiology and Molecular Genetics, University of thePunjab, Lahore, 54590 Pakistan; 2 Ittefaq Trust Hospital, Lahore, Pakistan

**Keywords:** Ventricular septal defects (VSDs), Pediatric patients, PCR-RFLP, Polymorphisms

## Abstract

**Background:**

Ventricular septal defects (VSDs) are malformations in the septum separating the heart’s ventricles. VSDs may present as a single anomaly (*isolated/nonsyndromic* VSD) or as part of a group of phenotypes (*syndromic* VSD). The exact location of the defect is crucial in linking the defect to the underlying genetic cause. The number of children visiting cardiac surgery units is constantly increasing. However, there are no representative data available on the genetics of VSDs in Pakistani children.

**Methods:**

Two hundred forty-two subjects (121 VSD children and 121 healthy controls) were recruited from pediatric cardiac units of Lahore. The clinical and demographic data of the subjects were collected. A total of four SNPs, one each from *MTRR, GATA4*, *VEGF*, and *ISL1* genes were genotyped by PCR-RFLP.

**Results:**

The results showed that the minor allele (T) frequency (MAFs) for the *MTRR* gene variant rs1532268 (c.524C > T) was 0.20 and 0.41 in the controls and the cases, respectively, with the genotype frequencies 3, 35, 62% in the controls and 12, 59 and 29% in the cases for TT, CT, CC genotypes, respectively (allelic OR: 5.73, CI: 3.82–8.61, *p-*value: 5.11 × 10^− 7^). For the *GATA4* variant rs104894073 (c.886G > A), the MAF for the controls and the cases was 0.16 and 0.37, respectively, the frequencies of AA, GA and GG genotypes were 2, 28, and 70% in the controls and 5, 64 and 31% of the cases (allelic OR: 3.08, CI: 2.00–4.74, *p*-value: 8.36 × 10^− 8^). The rs699947 (c.-2578C > A) of *VEGF* gene showed MAF 0.36 and 0.53 for the controls and cases, respectively, with the genotype frequencies 13, 42, and 45% in the controls and 22, 15, and 63% in the cases for the AA, CA, CC (allelic OR: 2.03, CI: 1.41–2.92, *p*-value: 0.0001). The *ISL1* gene variant rs6867206 (g.51356860 T > C), the MAFs were 0.26 and 0.31 in the controls and cases, respectively. The genotype frequencies were 48, 52, 0% in the controls and 39, 61, 0% in the cases for TT, TC, CC genotypes (allelic OR: 0.27, CI: 0.85–1.89, *p*-value: 0.227). The *MTRR*, *GATA4* and *VEGF* variants showed association while *ISL1* variant did not appear to be associated with the VSD in the recruited cohort.

**Conclusion:**

This first report in Pakistani children demonstrates that single nucleotide polymorphisms in genes encoding transcription factors, signaling molecules and structural heart genes involved in fetal heart development are associated with developmental heart defects., however further work is needed to validate the results of the current investigation.

**Supplementary Information:**

The online version contains supplementary material available at 10.1186/s13052-021-01022-7.

## Background

Congenital heart defects (CHDs) are the cardiovascular deformities that arise due to the abnormal development of heart during fetal development [1]. Globally, the estimated prevalence of CHD is about 8–10/1000 live births. Among all CHDs, ventricular septal defects (VSD) have the highest prevalence (1.5–3.5 per 1000 live births). VSDs account for 25–35% of all CHDs. Asia has the highest prevalence of CHD (9.3/1000 live births) and VSDs (2.62/1000 live births) [2]. In Pakistan, 30% of patients diagnosed with CHD have VSD [3]. VSDs result if the wall between the ventricular chambers of heart is not fully developed leaving a hole in the septum [4]. This malformation can manifest as single anomaly (known as isolated VSD), can be complex with intracardiac lesions, or can be a part of more complicated anomalies, for example tetralogy of Fallot (TOF), double outlet right ventricle etc. [5].

Dysregulation of the enzymes involved in homocysteine pathway due to single nucleotide polymorphisms plays an important role in causing VSD. Previous studies showed that periconceptional folic acid supplementation had a protective effect against embryonic developmental defects. The *MTRR* gene encodes the enzyme Methionine synthase reductase (MSR) which is involved in the folate metabolism/homocysteine pathway. Mutation in the gene encoding this enzyme can cause hyperhomocysteinemia, which is risk factor for heart disease. *MTRR* polymorphism e.g. rs1532268 (c.524C > T) is considered to be risk factor for the development of VSD [6, 7].

Many transcription factors (e.g. NKX2.5, GATA4, TBX5) and signaling pathway molecules are involved in normal heart development [8]. The GATA family (*GATA1–6*) comprises the zinc finger transcription factors that regulate the transcription of their respective genes by binding to the promoter at the DNA sequence GATA. *GATA4* is a highly expressed transcription factor during embryonic heart formation [9]. In 2003, mutations in *GATA4* gene were shown to result in VSD phenotype [10]. The chromosomal location of *GATA4* gene is 8p23.1 with length of 3371 bp, consisting of 6 exons [11]. A number of previous studies have shown association of the variant rs104894073 (c.886G > A) of exon 3 of GATA4 gene with VSD [12]. This variant reduces the affinity of the GATA4 transcription factor to bind with DNA causing reduction in its transcriptional activity [13].

Vascular endothelial growth factor (VEGF) is an angiogenic regulator and signaling protein involved in the formation of blood vessels. It also plays an important role in the development of the heart. Dysregulated VEGF plays an important role in the pathogenesis of VSD. Studies showed that SNP rs699947 (c.-2578C > A) located in the *VEGF* promoter was associated with increased risk for isolated VSD [14].

ISLET1 (encoded by the *ISL1 gene*) also plays an important role in cardiac morphogenesis. *ISL1* is present on chromosome 5q11.1 and encodes a transcription factor that controls the differentiation of secondary heart field. ISL1 progenitor cells produce almost two thirds of heart cells. SNP rs6867206 (g.51356860 T > C) is considered to be associated with CHD [15].

The aim of current study was to recruit the samples (blood/buccal swab) from Pakistani pediatric patients with isolated ventricular septal defect (VSD) and age/gender matched controls from the same population and investigate the selected SNPs 1) *MTRR*; NM_002454; rs1532268; c.524C > T; p.Ser175Leu, 2) *GATA4*; NM_001308093; rs104894073; c.886G > A; p.Gly296Ser, 3) *VEGF*; NM_003377; rs699947; c.-2578C > A, 4) *ISL1*; rs6867206; intergenic; g.51356860 T > C in the recruited cohort.

## Methods

### Study subjects

One hundred twenty-one blood/buccal swab samples of isolated VSD patients (72 males and 49 females) were collected from Ittefaq Hospital and Cardiology ward, Children’s Hospital, Lahore, Pakistan. One hundred twenty-one control blood samples (99 males and 22 females) were collected from children from different hospitals who did not have heart defects. The new children referred to the tertiary referral centers below 15 years of age were included in the study. A written informed consent was obtained from patients or guardians and the current study was approved by institutional ethical committee.

### Inclusion criteria

The diagnosis was made based on echocardiography. Size, number and exact location of the defect as well as magnitude of shunt were identified by two dimensional and Doppler echocardiography. Pulmonary artery pressure was estimated by using modified Bernoulli equation. Aortic valve prolapse and aortic regurgitation were also noted. Severity of aortic regurgitation was assessed by using parameters like left ventricular end-diastolic and systolic dimensions, doppler flow velocity measurement and assessment of length, width and area of regurgitant jet. All echocardiograms were performed by trained pediatric cardiologist. The samples having VSD with other cardiac or extracardiac symptoms (syndromic VSD) and any seropositive sample (for HBV/HCV/HIV) in both cases and controls were excluded from the study.

### Data collection of parameters and risk factors

Data was collected regarding age, family history, consanguineous marriage, maternal hemoglobin level during pregnancy, use of antibiotics during pregnancy etc. and other hematological parameters like White Blood Cell Count, Red Blood Cell Count, Hemoglobin level, Platelets count, Blood Urea Nitrogen (BUN), Serum Creatinine (SC), Calcium, Sodium, Potassium, Bilirubin, Serum glutamic pyruvic transaminase, Serum glutamic oxaloacetic transaminase, Alkaline Phosphate, Serum albumin and Gamma GT etc. were measured.

### Genotyping

Blood/buccal swab samples were taken and preserved at − 20 °C. Genomic DNA from the human leukocytes/epithelial cells was isolated using the salting out method. Genomic DNA quality was analyzed using a 0.9% agarose gel. The sequences of the primers used for the amplification of the *MTRR*, *GATA4*, *VEGF* and *ISL1* gene polymorphisms, their respective restriction enzymes and the digested fragment lengths are given in supplementary Table 1. PCR reaction conditions and RFLP digestion mixtures were optimized for each genetic marker. The PCR and RFLP products were run on1.5/2% agarose gel.

### Statistical analysis

Statistical Package for Social Sciences (SPSS version 22, IBM statistics) was used for statistical analysis. Mean and standard deviation were calculated for each parameter. Genotypic frequencies in cases and controls were calculated via chi-square (χ^2^) whereas allelic frequencies were calculated by direct count. Genotypic and allelic frequencies were reported as counts and percentages. The normality of all quantitative variables was checked via Shapiro–Wilk test. The study population was tested for Hardy-Weinberg equilibrium. Due to the inclusion of 4 SNPs, a Bonferoni adjusted *p* value of 0.0125 was used as significance threshold for all analyses.

## Results

The mean age (months) in cases was 20 ± 3.2, (mean age of males 20.8 ± 4.1 and of females 19.8 ± 5.5, range 1–169 months). 6% of patients had siblings affected with VSD while 18% had first degree relative affected with heart diseases. Parents of 67% patients were first cousins. The descriptive characteristics of hematological parameters have been described elsewhere [16].

In case of the rs1532268 (c.524C > T) variant of *MTRR* gene, the TT, CT and CC genotypes are 12, 59 and 29% respectively among cases versus 3, 35 and 62% respectively among controls. The allelic frequency of T and C are 0.41 and 0.59 respectively, among cases, versus 0.20 and 0.80 respectively, among controls. The genotype distribution in dominant and recessive models are given in Table 1 (Dominant; OR: 0.25, CI: 0.15–0.43, *p*-value: 2.4 × 10^− 7^, Recessive: OR: 5.15 CI: 1.44–18.40, *p*-value: 0.005, allelic OR: 5.73, CI: 3.82–8.61, *p*-value: 5.11 × 10^− 7^). The genotype frequencies were in Hardy-Weinberg equilibrium (*p*-value: 0.07).

**Table 1:** Allele and genotype frequencies of the selected SNPs in the study cohort

SNP (Gene)	Model	Genotype	Cases	Controls	OR (CI), *p*-value
rs1532268 (c.524C > T) (*MTRR*)	Dominant	CT + TT CC	86 35	75 46	0.25 (0.15–0.43), 2.4 × 10^−7^
	Recessive	TT CT + CC	14 107	3 118	5.15 (1.44–18.40), 0.005
	Alleles	T C	0.41 0.59	0.2 0.8	5.73 (3.82–8.61), 5.11 × 10^−7^
rs104894073 (c.886G > A) (*GATA4*)	Dominant	GA + AA GG	84 37	85 36	0.19 (0.11–0.32), 6.7 × 10^−10^
	Recessive	AA GA + GG	6 115	2 119	3.0 (0.61–15.70), 0.15
	Alleles	A G	0.37 0.63	0.16 0.84	3.08 (2.00–4.74), 8.36 × 10^− 8^
rs699947 (c.-2578C > A) (*VEGF*)	Dominant	CA + AA CC	103 18	71 50	0.25 (0.13–0.46), 4.7 × 10^− 6^
	Recessive	CA + CC AA	95 26	105 16	1.80 (0.90–3.55), 0.089
	Alleles	A C	0.53 0.47	0.36 0.64	2.03 (1.41–2.92), 0.0001
rs6867206 (g.51356860 T > C) (*ISL1*)	Dominant	TC + CC TT	74 47	64 57	0.71 (0.43–1.19), 0.194
	Recessive	TC + TT CC	121 0	121 0	–
	Alleles	C T	0.31 0.69	0.26 0.74	0.27 (0.85–1.89), 0.227

For the rs104894073 (c.886G > A) variant of GATA4, the percentage of AA, GA and GG genotypes are 5, 64 and 31%, respectively among cases, versus 2, 28 and 70% among controls. The allelic frequency of A and G are 0.37 and 0.63 respectively, among cases, versus 0.16 and 0.84 respectively, among controls (OR: 3.08, CI: 2.00–4.74, *p*-value: 8.36 × 10^− 8^). In the dominant model, the OR was 0.19 with CI 0.11–0.32 and *p*-value 6.7 × 10^− 10^ while in the recessive model, the OR was 3.0 with CI 0.61–15.70 and *p*-value 0.15. The genotype frequencies were in Hardy-Weinberg equilibrium for the control group (*p*-value: 0.49).

The genotyping of rs699947 (c.-2578C > A) variant of *VEGF* gene showed that the percentages of AA, CA and CC genotype were 22, 15, and 63% respectively among cases versus 13, 42, and 45% among controls. The allelic frequency of A and C are 0.36 and 0.64 respectively, among cases, versus 0.54 and 0.47 respectively, among controls (allelic OR: 2.03, CI: 1.41–2.92, *p*-value: 0.0001). The genotype distribution in dominant and recessive models are given in Table 1 (Dominant; OR: 0.25, CI: 0.13–0.46, *p*-value: 4.7 × 10^− 6^, Recessive: OR: 1.80 CI: 0.90–3.55, *p*-value: 0.089). The genotype frequencies were in Hardy-Weinberg equilibrium for the control group (*p*-value: 0.886).

For the *ISL1* variant rs6867206 (g.51356860 T > C), the percentages of CC, TC and TT genotypes were 0, 61 and 39%, respectively among cases, versus 0, 52 and 48% among controls. The allelic frequency of C and T alleles are 0.31 and 0.69 respectively, among cases, versus 0.26 and 0.74 respectively, among controls (allelic OR: 0.27, CI: 0.85–1.89, *p*-value: 0.227). In the dominant model, the OR was 0.71 with CI 0.43–1.19 and *p*-value 0.194 while the recessive modelcould not be executed due to absence of homozygous recessive genotype in the cases as well as the controls (Table 1). The genotype frequencies deviated from Hardy-Weinberg equilibrium for the cohort (*p*-value: 0.0001).

## Discussion

The current study aimed to genotype the variants in the genes involved in various stages of cardiac development. This is very important especially for the developing world where the management of developmental heart defects is costly and corrective surgery is unaffordable for many families. Globally, the advancements in the treatments available for septal defects have resulted in an increase in life span from just few years of life to adulthood. Underpinning the genetic causes can be significant for the design of a prenatal screening of at-risk families and genetic counseling. The distinct genetic architecture of Asian populations is a geneticist’s dream to investigate the genetic markers for a large number of genetic diseases; however this bears the price of high prevalence of genetic disorders in consanguineous populations.

For rs1532268 (c.524C > T) variant of *MTRR* gene, there was a difference in frequencies of C and T alleles, and this difference was statistically significant. This result is in consensus with a previous study [6] whereby *MTRR* gene variant rs1532268 (c.524C > T) has been reported as a potential risk factor for the development of VSD in Iranian subjects. As homocystein metabolism is very important in cardiac development, the higher proportion of risk genotypes in the case group is an indication of a possible effect on heart development.

GATA family of transcription factors controls the growth of many tissue types, GATA4 is a highly expressed transcription factor during cardiac embryogenesis. The *GATA4* gene if mutated can lead to changes in the signaling during heart formation process. In case of rs104894073 (c.886G > A) variant of *GATA4* gene, the GG genotype was more prevalent in the controls (70%) than the cases (31%). The frequency of AA genotype is more prevalent in cases (5%) than in controls (2%). Previous studies [17] failed to find any association between *GATA4* gene variant rs104894073 (c.886G > A) with VSD. This variant is rare and its frequency has been reported to be very low previously. The original investigation reporting this variant identified it in familial cases of VSD but not in the control subjects [18]. The relatively high frequency of this variant observed in the current cohort can be attributed to ethnic differences or sample bias.

The formation of accurate vasculature is a critical factor shaping the final heart structures and chambers. An alteration in this process may lead to cardiac malformation including structural defects. The vasculature is largely affected a number of growth regulators, an important of which is vesicular endothelial growth factor (VEGF). Variations in the gene coding for this protein can lead to a number of consequences. The selected rs699947 (c.-2578C > A) variant of *VEGF* gene showed a statistically significant difference in the frequencies of C and A alleles between the cases and the controls. The higher frequency of risk allele in the case cohort may have been due to the underlying effect on heart development. Although these effects do not show causation, they are important in establishing associations between the low-modest effect size variants and the outcome.

In case of rs6867206 (g.51356860 T > C) variant of *ISL1* gene, we could not detect any association between the variant and the outcome. The homozygous polymorphic genotype was absent in the cases and the controls. The dominant and allelic models also did not prove any relation between this variant and VSD. The minor allele frequencies were slightly different between the cases and the controls and this variant did not appear to be a modest effect size variant contributing to affect the heart development in the recruited cohort.

## Conclusion

We describe the findings of genotyping the polymorphisms in the *MTRR*, *GATA4*, *VEGF* and *ISL1* genes in Pakistani children with isolated ventricular septal defects. This is a unique population in which consanguineous marriages combined with religious, social and cultural stratifications, play an important role in the development of a number of genetic diseases. The study had the limitations of relatively small sample size, inclusion of a limited number of genetic markers and samples coming from one province of Pakistan only. Despite these limitations, the current report represents the results of the first preliminary attempt to identify the genetic basis of ventricular septal defects in the Pakistani children.

## Supplementary Information

**Additional file 1: Supplementary Table 1.** Primers, enzymes, products and digestion fragment sizes for the selected variants.

## Data Availability

All the necessary information has been provided along with the manuscript, however, the corresponding author can be contacted for any information related to this paper.

## Abbreviations

VSD: Ventricular septal defects
MTRR: 5-Methyltetrahydrofolate-Homocysteine Methyltransferase Reductase
VEGF: Vascular endothelial growth factor
ISL1: ISLET1
CHD: Congenital heart defects
SPSS: Statistical Package for Social Sciences
