# Supplementary material for: First report of polymorphisms in MTRR, GATA4, VEGF, and ISL1 genes in Pakistani children with isolated ventricular septal defects (VSD)
Source: Ital J Pediatr. 2021 Mar 23;47:70. doi: 10.1186/s13052-021-01022-7 (PMC7989246; doi:10.1186/s13052-021-01022-7)
Supplement: Supplementary file 1 — Additional file 1: Supplementary Table 1. Primers, enzymes, products and digestion fragment sizes for the selected variants. [file 13052_2021_1022_MOESM1_ESM.docx]

Supplementary Table 1: Primers, enzymes, products and digestion fragment sizes for the selected variants

| **SNPs** | **Gene** | **Primers sequence** | **PCR product** | **Restriction Enzyme** | **Restriction Fragment Size** |
| --- | --- | --- | --- | --- | --- |
| rs104894073 (c.886G>A) | GATA4 | F-5’-GAGTGGGCCTCTCCTGTG-3’  R-5’-GAGAGATGGGCATCAGAAGG-3’ | 166bp | *PstI* | Wild type: 166bp  Heterozygote: 166bp, 90bp,76bp  Mutant: 90bp, 76bp |
| rs1532268 (c.524C>T) | MTRR | F-5'-GTCAAGCAGAGGACAAGAG-3'  R-5'AGAGACTCCTGCAGATGTAC-3' | 309bp | *XhoI* | Wild type: 251bp, 58bp  Heterozygote: 309bp, 251bp, 58bp  Mutant: 309bp |
| rs6867206 (g.51356860T>C) | ISL1 | F-5’-ACTGCTGCAAAATTCCAACC-3’  R-5’-AGAGGGCCCATAGGTCTGAT-3’ | 296bp | *BgIII* | Wild type:296  Heterozygote:296, 216, 80  Mutant: 216, 80 |
| rs699947 (c.-2578C>A) | VEGF | F-5’-GAGGATGGGGCTGACTAGGT-3’  F-5’-TGGTTTCTGACCTGGCTATTTC-3’ | 229bp | *AciI* | Wild type:125,104  Heterozygote:229,125,104  Mutant: 229 |
